# Cyclodextrin-Based Magnetic Nanoparticles for Cancer Therapy

**DOI:** 10.3390/nano8030170

**Published:** 2018-03-16

**Authors:** Radosław Mrówczyński, Artur Jędrzak, Kosma Szutkowski, Bartosz F. Grześkowiak, Emerson Coy, Roksana Markiewicz, Teofil Jesionowski, Stefan Jurga

**Affiliations:** 1 NanoBioMedical Centre, Adam Mickiewicz University in Poznan, Umultowska 85, PL-61614 Poznan, Poland; artur.jedrzak@gmail.com (A.J.); bartoszg@amu.edu.pl (B.F.G.); coyeme@amu.edu.pl (E.C.); rokmar@amu.edu.pl (Ro.M.); stjurga@amu.edu.pl (S.J.); 2 Faculty of Chemical Technology, Institute of Chemical Technology and Engineering, Poznan University of Technology, Berdychowo 4, PL-60965 Poznan, Poland; teofil.jesionowski@put.poznan.pl

**Keywords:** magnetic nanoparticles, polydopamine, theranostic, MRI, nanomedicine

## Abstract

Polydopamine (PDA)-coated magnetic nanoparticles functionalized with mono-6-thio-β-cyclodextrin (SH-βCD) were obtained and characterized by transmission electron microscopy (TEM), Fourier transform infrared spectroscopy (FT-IR), X-ray photoelectron spectroscopy (XPS), Nuclear and Magnetic Resonance Imaging (NMR and MRI), and doxorubicin (DOXO)-loading experiments. The liver cancer cellular internalization of DOXO-loaded nanoparticles was investigated by confocal imaging microscopy. Synthesized nanomaterials bearing a chemotherapeutic drug and a layer of polydopamine capable of absorbing near-infrared light show high performance in the combined chemo- and photothermal therapy (CT-PTT) of liver cancer due to the synergistic effect of both modalities as demonstrated in vitro. Moreover, our material exhibits improved *T*_2_ contrast properties, which have been verified using Carr-Purcell-Meiboom-Gill pulse sequence and MRI Spin-Echo imaging of the nanoparticles dispersed in the agarose gel phantoms. Therefore, the presented results cast new light on the preparation of polydopamine-based magnetic theranostic nanomaterials, as well as on the proper methodology for investigation of magnetic nanoparticles in high field MRI experiments. The prepared material is a robust theranostic nanoasystem with great potential in nanomedicine.

## 1. Introduction

The development of nanotechnology has enabled a breakthrough in the synthesis of modern drug nanocarriers. Significantly, the delivery and bioavailability of encapsulated hydrophobic drugs to the tumor cells have been greatly improved thanks to the nanoengineered drug carriers [1,2,3]. A variety of reaction paths are available nowadays and the surface of nanomaterials can be tailored on demand. That includes targeted functionalization by functional ligands and complexes [4,5], PEG polymer chains [6], fluorescent dyes etc., which can be bonded to the nanomaterials in a relatively straightforward manner resulting in a new class of advanced nanocarriers capable of bimodal functionality, such as simultaneous therapy and diagnosis (theranostics) [7,8,9,10]. 

The most common class of materials used in the synthesis of theranostic nanostructures are carbon nanotubes, graphene, up-converting nanoparticles, and gold nanostructures [11,12,13,14,15,16,17]. However, the nanotoxicity, complex and time-consuming synthetic procedures, and poor stability are the predominant barriers for the clinical application of these nanomaterials. Therefore, there is still a challenge to develop new biocompatible nanostructures with multifunctional capabilities, especially for liver cancer which is one of the most frequent types of cancer and the leading cause of cancer deaths worldwide, responsible for more than 600,000 deaths each year. The use of magnetic nanoparticles (MNPs) as a base for theranostic nanomaterials has multiple advantages over other types of nanomaterials. First of all, they can be used as contrast agents in MRI [18,19,20]. Furthermore, they can be guided by an external magnetic field to increase the drug concentration in a tumor, which results in higher treatment efficiency [21]. Consequently, the side effects of chemotherapy can be diminished and patients’ comfort level improved. In addition, MNPs are non-toxic and biocompatible and approved by the FDA [22]. All of these render them as an ideal material for building advanced theranostic tools. Polydopamine (PDA) is a novel polymer with uniquely strong adhesive properties, which can be deposited on either hydrophilic or hydrophobic surfaces. The reaction of polymerization is based on the oxidative polymerization of dopamine under basic conditions [23,24,25,26,27,28,29]. As a matter of fact, PDA was often used to coat different classes of nanostructures including magnetic nanomaterials [30]. Recent studies on cyto- and genotoxicity of PDA-coated magnetic nanomaterials show that PDA coating is biocompatible and does not cause any damage to genetic material [31]. Lately PDA-coated magnetic nanomaterials have been used in the thermal ablation of cancer due to the intrinsic photothermal properties of PDA [32,33,34]. Such an approach towards cancer treatment seems to work quite effectively, however, there are still some drawbacks of photothermal therapy (PTT). For instance, due to light scattering and limited absorption in tumoral tissues, it is cumbersome to fully remove solid tumors using only PTT [35]. On the other hand, the model chemotherapeutic drug doxorubicin (DOXO) was previously deposited on magnetic nanoparticles, although moderate loading capacity was obtained and relatively high dosage was required in order to obtain good performance in the anticancer therapy [30]. Furthermore, the potential applications as an MRI contrast agent have not yet been explored.

Cyclodextrins (CDs) are compounds that consist of at least 5 d-glucopyranoside units bound together in a toroidal shape linked by the α-1,4-acetal bond [36]. CDs have drawn attention as potential drug carriers for hydrophobic drugs [37,38]. The outer surface of CDs is hydrophilic, while the internal cavity is hydrophobic and thus perfectly suitable for containing hydrophobic drug molecules. A lot of effort has been made to link β-cyclodextrins with nanomaterials in order to obtain functionalized nanostructures with improved properties for biomedicine. For example, the combination of β-cyclodextrin with MNPs gave rise to nanocarriers with high loading capacity capable of delivering curcumin [39], ketoprofen [40], or phenylacetic acid to different cancer cells with good efficacy [41]. An exhaustive review of this topic was recently published by Bina Gidwani and Amber Vya [42]. As a matter of fact, just recently the application of PDA-coated magnetic nanoparticles with cyclodextrins for delivery of diclofenac was reported [43]. Nevertheless, the application of CDs in preparation of advanced nanocarriers with bimodal functionality and contrast properties still remains an unexplored area.

Here we present a new magnetic nanoplatform based on Fe_3_O_4_@PDA@SH-βCD, with high loading capacity due to the presence of βCD moieties in the structure, for combined chemo- and photothermal therapy of liver cancer, along with profound research on their contrast properties both in water and agarose gel matrix by nuclear and magnetic resonance imaging. Moreover, we prove that immobilization of obtained nanostructures has a significant influence on their contrast properties and seems to be a more accurate methodology in the determination of contrast properties of synthesized materials. The presented data are of great importance for the field of multifunctional magnetic nanomaterials covered with PDA for a theranostic approach toward liver cancer and fill the gap in the literature regarding their proper characterization by Nuclear and Magnetic Resonance Imaging (NMR and MRI).

## 2. Materials and Methods

All of the chemical reagents were purchased from Sigma-Aldrich (Poznan, Poland), except for the dopamine hydrochloride (Alfa Aesar, Gdansk, Poland) and doxorubicin hydrochloride (LC Laboratories, Boston, MA, USA). All reagents and solvents were of reagent-grade quality. For all experiments, Milli-Q deionized water (resistivity 18 MΩ·cm^−1^) was used. 6-thio-β-cyclodextrin (SH-βCD) was kindly donated by CycloLab, (Budapest, Hungary).

### 2.1. Synthesis of Fe_3_O_4_@PDA (Nanomaterial A) and Fe_3_O_4_@PDA@SH-βCD (Nanomaterial B)

In order to obtain Fe_3_O_4_@PDA nanoparticles (nanomaterial A), the FeCl_2_·4H_2_O (1.7 g, 8.57 mmol) and FeCl_3_·6H_2_O (4.7 g, 16.2 mmol) were dissolved in 80 mL of deionized water. Then, the solution was degassed with nitrogen and heated up to 90 °C, followed by the addition of 20 mL of 25% ammonia aqua. The heating was continued for 30 min and then 8 mL of 20% citric acid was added. The mixture was kept for 1.5 h at 90 °C. Finally, the magnetic nanoparticles were collected by an external magnetic field and rinsed with deionized water. 200 mg of the obtained nanoparticles were transferred to a flask containing 400 mL of tris(hydroxymethyl)aminomethane buffer (TRIS) (pH = 8.5, 10 mM). The dopamine coating was governed by the addition of dopamine hydrochloride (200 mg, 1.055 mmol) followed by subsequent stirring for 6 h. Finally, PDA-coated nanoparticles were again collected by an external magnet and washed with water.

Nanomaterial B (Fe_3_O_4_@PDA@SH-βCD) was obtained from Fe_3_O_4_@PDA nanoparticles using thio-Michael reaction between 6-thio-β-cyclodextrin and quinone groups present in PDA structure. Nanoparticles (100 mg) were mixed with 6-thio-β-cyclodextrin (500 mg, 0.43 mmol) in 200 mL of TRIS buffer (pH = 8.5) for 4 h with magnetic stirring at 25 °C. The obtained nanoparticles were collected by an external magnet and purified with deionized water and suspended in 20 mL of Milli-Q water.

### 2.2. Nuclear and Magnetic Resonance Imaging (NMR and MRI) Studies

#### 2.2.1. Transverse Relaxation *T*_2_ for Fe_3_O_4_@PDA in Water Suspensions

Transverse relaxation times *T*_2_ were obtained in water suspensions using Agilent DD2 600 MHz spectrometer (Santa Clara, CA, USA) and DOTY DSI-1374 1H diffusion probe. Due to an immanent lack of stabilization in the magnetic field, we decided to perform an initial characterization of water suspensions using an NMR spectrometer (Columbia, SC, USA) equipped with a pneumatic lift so that the total experimental time could be shorter than 1 min. The transverse relaxation rates of water protons *R*_2_ = 1/*T*_2_ were obtained using Fourier transform Carr-Purcell-Meiboom-Gill pulse sequence: sequence (π/2)x − [(τ_cp_/2) − πy − τ_cp_/2 − spin echo]*n*. The π/2 − π delay τ_cp_ was set to 1 and 2 ms, and the total number of acquired spin echoes *n* was 8. The relaxation delay was 2.5 s with two dummy scans. Accordingly, the total experimental time was 34 s. The temperature was set to 295 K. The experiment was performed with only one accumulation in order to keep the experimental time as short as possible and thus not diminish the effects of the sample precipitation. Prior to the experiment each sample was stirred for 2 min.

#### 2.2.2. Transverse Relaxation *T*_2_ in Agarose Gels from Multi-Echo Multi-Slice MEMS MRI

The experimental setup for magnetic resonance imaging studies consisted of an Agilent 9.4 T MRI preclinical scanner equipped with a 120 mm gradient coil (2 mT/m/A) and 30 mm millipede coil. In order to perform a quantitative *T*_2_ relaxation experiment (at 18 °C) we employed MEMS protocol. Each echo is acquired after an excitation pulse with an increasing echo time. The parameters of the data acquisition were as follows: field of view 15 mm (FOV), matrix size 128 × 128, Gaussian-shaped pulse with 2048 µs length. The echo time was set to 4 and 10 ms in two separate experiments. In total, 16 consecutive images with varied echo time were acquired for each sample. The initial analysis was performed in VnmrJ 4.2 revision software (Santa Clara, CA, USA). The raw relaxation data points were collected from an average intensity obtained from circular Region of Interests (ROIs) and then analyzed using Origin 8.5 software (Origin Lab, Northampton, MA, USA) using simple single exponential decay function. In order to prepare MRI agarose-based phantom, nanoparticles B were suspended in a hot 2% agarose solution. The hot solution was finally transferred to 10 mm plastic vials and left for full agarose gelation.

### 2.3. FT-IR, UV-VIS, XPS, TGA, Zeta Potential, DLS and Magnetic Measurements

FT-IR spectra were collected using a Vertex 70, (Billerica, MA, USA) in KBr pellets. UV-Vis measurements were performed on a Perkin-Elmer Lambda 950 UV/Vis/NIR (Waltham, MA, US). X-ray photoelectron spectroscopy analysis was performed on a SAGE HE100b-SPECS (Berlin, Germany), with a Mg source (Kα 1283.6 eV). Samples were dried out and the powders were placed in a multipurpose high vacuum holder. Thermogravimetric analysis was performed on Jupiter STA 449F3 Netzsch apparatus (Selb, Germany). Dynamic light scattering (DLS) and zeta potential measurements were carried out on Zetasizer Nano-ZS ZEN 3600 produced by Malvern Instruments Ltd. (Malvern, UK) using DLS mode or Laser Doppler Electrophoresis, respectively. Magnetic measurements were carried out in the temperature range 2–300 K in magnetic fields up to 5 T using a Quantum Design MPMS SQUID magnetometer (San Diego, CA, USA). Temperature dependence of magnetization *M*(T) was measured in zero field cooling (ZFC) and field cooling (FC) mode in a magnetic field of 1 kOe. Magnetization curves *M*(H) were obtained at 5 and 300 K.

### 2.4. TEM and Confocal Microscopy Imaging

Transmission electron microscopy (TEM) images were recorded on a JEM-1400 microscope made by JEOL (Tokyo, Japan). The accelerating voltage was 120 kV. A small amount of the sample was placed on a copper measuring grid (Formvar/Carbon 200 mesh made by TedPella (Redding, CA, USA) after 5 min of sonication in deionized water. For visualization under a confocal microscope, the HepG2 cells after 24 h incubation with Fe_3_O_4_@PDA@SH-βCD@DOXO were fixed with 3.7% formaldehyde (Sigma-Aldrich, Poznan, Poland) in PBS buffer (10 mM, pH = 7.4) for 15 mins. The cell nuclei were stained with Hoechst 33342 (Molecular Probes, Eugene, OR, USA) at a concentration of 8 µM. Cells were imaged using a confocal laser scanning microscope (Olympus FV1000, Tokyo, Japan). Image acquisition and analysis were performed with a 60× objective, a 1.4 oil immersion lens, and FV10-ASW software (Olympus, Tokyo, Japan). Images of the Fe_3_O_4_@PDA@SH-βCD@DOXO were visualized using 488 nm excitation and 560–590 nm emission filters. The Hoechst fluorescence was detected using 405 nm excitation source and 425–472 nm emission filters. 

### 2.5. Loading and Release of Doxorubicin from Fe_3_O_4_@PDA@SH-βCD (Nanomaterial B + DOXO)

2 mg of nanomaterial B were mixed with 2 mL of DOXO solution in PBS buffer at concentration 1 mg/mL. The mixture was shaken at 23 °C for 24 h. The resulting nanoparticles Fe_3_O_4_@PDA@SH-βCD@DOXO were collected by an external magnet and washed twice with PBS buffer. The amount of the linked doxorubicin in supernatant was analyzed by UV-Vis spectroscopy at 486 nm. The release of DOXO from nanomaterial B was performed according to the following procedure. 2 mg of Fe_3_O_4_@PDA@SH-βCD@DOXO was mixed with citric buffer (2 mL, 10 mM) at pH 4.5 and 5.5, respectively. Then, the mixture was shaken at 37 °C and the sample was collected at appropriate time intervals, refilling the mixture with the fresh portion of the buffer. The amount of doxorubicin released from the carrier was determined by standard calibration curve method by UV-Vis spectroscopy at 486 nm.

### 2.6. NIR Laser Irradiation of Fe_3_O_4_@PDA@SH-βCD (Nanomaterial B)

Concentrations of Fe_3_O_4_@PDA@SH-βCD nanoparticles in water were varied between 0 and 0.1 mg/mL. The solution was placed into a quartz cuvette (total volume of 1 mL) and irradiated with a NIR laser at 808 nm wavelength and average power at 2 W/cm^2^ (Changchun New Industries Optoelectronics Tech. Co., Ltd., Changchun, China). The temperature of the solutions was measured by a digital thermometer with a thermocouple sensor. Additionally, thermal imaging was performed using a SONEL KT-160 thermal camera (Swidnica, Poland).

### 2.7. Cytotoxicity Assays

HepG2 cells were purchased from American Type Culture Collection (ATCC) and cultured in a Minimum Essential Medium Eagle (MEM, Sigma-Aldrich, Poznan, Poland) medium, supplemented with 10% Fetal Bovine Serum (FBS, Sigma-Aldrich, Poznan, Poland), 1% antibiotics (penicillin 100 µg/mL, streptomycin 100 µg/mL, Sigma-Aldrich, Poznan, Poland), non-essential amino acids (Sigma-Aldrich, Poznan, Poland), and sodium pyruvate (Sigma-Aldrich, Poznan, Poland) under standard conditions (37 °C, 5% CO_2_). A WST-1 cell proliferation assay was carried out to assess the cytotoxicity of the magnetic nanoparticles. The WST-1 Cell Proliferation Assay (Clontech, Fremont, CA, USA) is based on the enzymatic cleavage of the tetrazolium salt WST-1 to a water-soluble formazan dye, which can be quantified by absorbance at 420–480 nm, by live cells. HepG2 cells were seeded at a density of 3 × 10^4^ cells per well in a 96-well plate. After 24 h, an increasing concentration from 0.5 up to 40 µg/mL of nanomaterials A and B were added to each well, and the cells were incubated for 24 h. Then, 10 µL of the WST-1 Cell Proliferation Reagent was added to each well and incubated for 4 h. After this time, the absorbance at 450 nm (reference wavelength 620 nm) was recorded against the background control, using a multiwell plate reader (Zenyth, Biochrom, Cambridge, UK) and the cell viability was expressed as the respiration activity normalized to untreated cells. All experiments were carried out in triplicate. It is important to highlight that the nanomaterials concentration used in the biological experiments refers to concentration of only nanocarriers without DOXO. Furthermore, the concentration of free DOXO corresponds to the amount of drug loaded on nanomaterial B.

### 2.8. Combined Chemo- and Photothermal Therapy

For photothermal therapy, HepG2 cells in 96-well plates were incubated with increasing concentration from 0.5 up to 40 µg/mL of nanomaterial B and DOXO-loaded nanomaterial B. After 4 h of incubation, the cells were irradiated by an 808 nm laser (Changchun New Industries Optoelectronics Tech. Co. Ltd., Changchun, China) with power density at 2 W/cm^2^ for 5 min. The cells were further incubated for 24 h, and the WST-1 cell viability assay was performed.

In order to determine the influence of a laser beam on the cytotoxicity of HepG2 cells, we have carried out the LIVE/DEAD assay. HepG2 cells at a density of 1.8 × 10^5^ cells per well (24-well plate) were incubated with a concentration of 40 µg/mL of nanomaterial B. The image acquisition was carried out using IN Cell Analyzer 2000 (GE Healthcare Life Sciences, Pittsburgh, PA, USA). The collected images were analyzed by IN Cell Developer Toolbox Image Analysis Software (Pittsburgh, PA, USA). After 4 h of incubation, the HepG2 cells were irradiated for 5 min with 808 nm laser at 2 W/cm^2^ power density. After 24 h incubation, the dead and living cells were further stained with 2 µM EthD-1 and 2 µM Calcein AM, respectively (ThermoFisher Scientific, Waltham, MA, USA).

## 3. Results and Discussion

### 3.1. Characterization of Fe_3_O_4_@PDA and Fe_3_O_4_@PDA@SH-βCD

**Figure 1:**
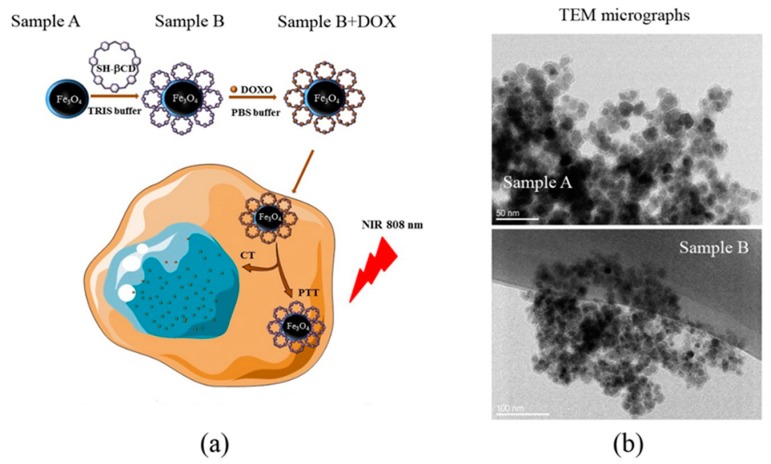
(**a**) The schematic representation of samples preparation. Sample A: Fe_3_O_4_@PDA. Sample B: Fe_3_O_4_@PDA@SH-βCD. Sample B + DOXO corresponds to Sample B loaded with doxorubicin for combined chemo- and photothermal therapy; (**b**) Transmission electron microscopy (TEM) micrographs of the synthesized nanoparticles. Sample A scale bar 50 nm, Sample B 100 nm.

The schematic representation of the synthesis of Fe_3_O_4_@PDA (nanomaterial A) and Fe_3_O_4_@PDA@SH-βCD (nanomaterial B) is shown in Figure 1a. It is worth highlighting that nanomaterial B with loaded doxorubicin is depicted in the text as Fe_3_O_4_@PDA@SH-βCD@DOXO. 

The TEM micrographs of nanoparticles A and B are shown in Figure 1b. Both samples were spherical in shape and their diameter was in a range of 8–14 ± 2 nm. A partial aggregation was observed, albeit the nanoparticles maintained their spherical morphology and uniform size distribution. PDA layer thickness was estimated to be around 2 to 3 nm (see Supplementary Materials Figures S1 and S2).

Partial aggregation was also observed in DLS measurements (Supplementary Materials Figure S3). Nevertheless, the hydrodynamic diameter after one week was not changed significantly (Supplementary Materials Table S1).

**Figure 2:**
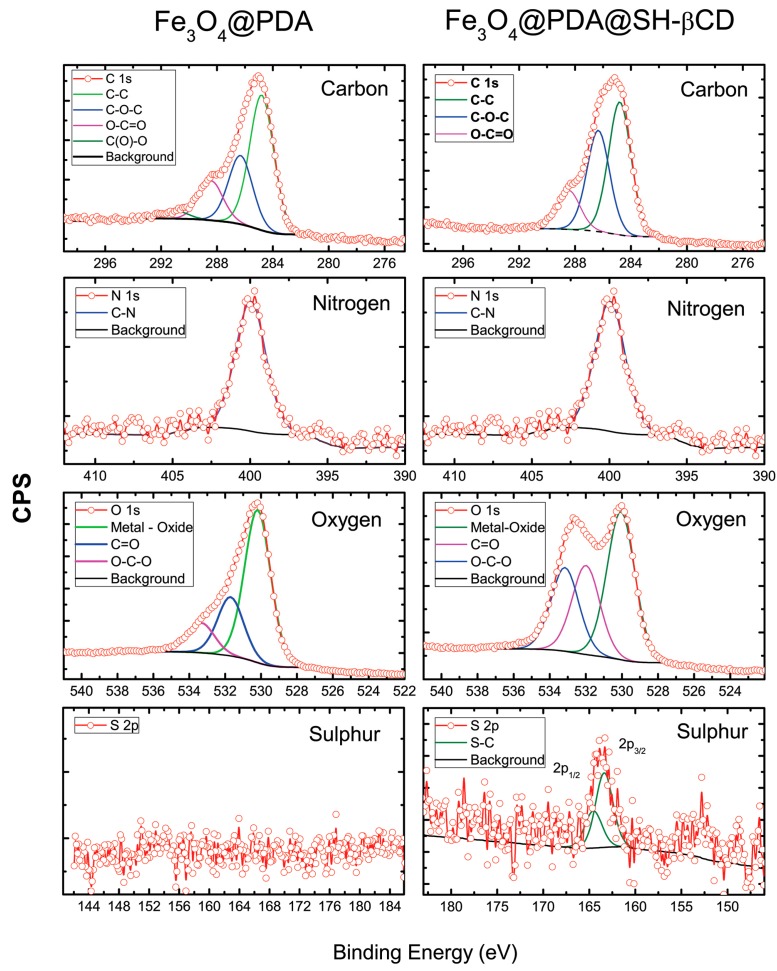
High-resolution XPS spectra for Fe_3_O_4_@PDA (sample A) and after functionalization with SH-βCD (Sample B).

The initial chemical composition of the obtained nanoparticles B was confirmed by XPS analysis. The corresponding XPS spectra are shown in Figure 2. The ’N 1s’ spectrum revealed only one peak at 399 eV which is assigned to the N–H/C–N bonds present in the polydopamine structure for both samples [44]. The deconvolution of the ‘C 1s’ spectrum shows three main contributions in the range between 284.8 to 288.4 eV which are assigned to C–C, C–OH/C–N, and C=O bonds from PDA molecules attached to SH-βCD. The ‘O 1s’ spectrum exhibited three components assigned to the oxygen atoms of the O–C–O group (533.2 eV), C=O groups (531.9 eV) and to metal oxides from Fe-O/metal (support) at around 530.0 eV [45]. The ‘S 2p’ spectrum of sample B exhibited only one specimen, with the main sulfur 2 p3/2 peak at around 163.4 eV which can be assigned to C–S–C bonds. Moreover, a decreased atom percentage of nitrogen and an increased intensity of carbon were observed in material B in comparison to material A.

**Figure 3:**
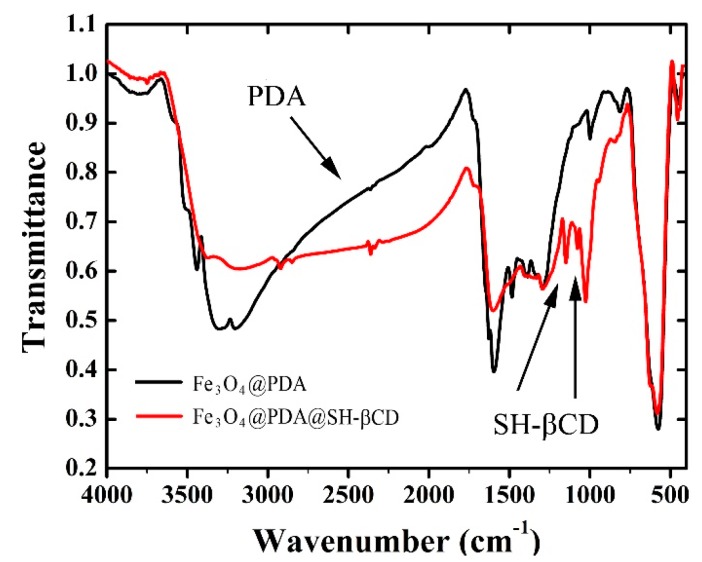
Fourier transform infrared spectroscopy (FT-IR) spectra obtained for Fe_3_O_4_@PDA and Fe_3_O_4_@PDA@SH-βCD nanoparticles.

Additionally, samples A and B were investigated by FT-IR (Figure 3). The characteristic band for PDA is visible for both samples in a range of 1440–1620 cm^−1^. The strong peak at 586 cm^−1^ was assigned to the Fe–O bond. Two new peaks in the recorded spectra of sample B were identified at 1078 cm^−1^ and 1152 cm^−1^, and assigned to C–O and C–O–C bonds from SH-βCD, respectively. The signals at 3160 to 3300 cm^−1^ observed in the spectra of A became broader and moved to 2960 to 3400 cm^−1^ with a maximum at ~3100 cm^−1^.

The amount of SH-βCD on the surface of the sample B was determined using thermogravimetric analysis (TGA) (Supporting Materials Figure S4). Both nanomaterials A and B showed a small weight loss below 150 °C which was attributed to the evaporation of water. Correspondingly, the TGA curve obtained for the Fe_3_O_4_@PDA sample revealed a mass loss of 18% in the temperature range between 200 and 750 °C, which was attributed to the thermal decomposition of the PDA layer. Similarly, in the same temperature region the weight loss for Fe_3_O_4_@PDA@SH-βCD was around 14%. The increased amount of the decomposed material in Fe_3_O_4_@PDA@SH-βCD compared to the initial Fe_3_O_4_@PDA nanoparticles confirms the functionalization with SH-βCD. Accordingly, we estimate the total concentration to the grafted SH-βCD within the bulk Fe_3_O_4_@PDA@SH-βCD to 4%.

With attention to the surface charge properties, the ζ-potential was measured for the obtained nanomaterials. Our results indicate that ζ-potential of bare magnetite nanoparticles was approximately −46 mV. After the coating with a PDA layer, the ζ-potential increased to −26 mV and after addition of SH-βCD moiety it changed down to −27 mV (Supporting Materials Figure S5).

The magnetic properties were studied using SQUID. The results are shown in Supplementary Materials Figure S6. The saturation magnetization (*M*s) of sample A was 56 emu/g. The lack of hysteresis loop confirmed the superparamagnetic character of both samples A and B. The saturation of magnetization determined for nanomaterial B was around 49 emu/g, which is in agreement with the results previously reported by other authors for Fe_3_O_4_@PDA nanoparticles [43]. Accordingly, the nanoparticles were easily controlled by an inhomogeneous magnetic field. The decrease of *M*s for Fe_3_O_4_@PDA@SH-βCD with respect to Fe_3_O_4_@PDA is caused by an increased organic shell whose thickness is increased due to the linkage of the SH-βCD to the surface of Fe_3_O_4_@PDA@SH-βCD.

### 3.2. Transverse Relaxation T_2_ in Fe_3_O_4_@PDA in Water 

The simplest method of estimating the transverse relaxation rates is to determine the line widths at half height. While the typical line width for water doped with D_2_O is around 3 Hz, the spectra for Fe_3_O_4_@PDA in water suspension were broad and inhomogeneous, with typical line widths at half height (FWHM) varied between 240 and 380 Hz.

**Figure 4:**
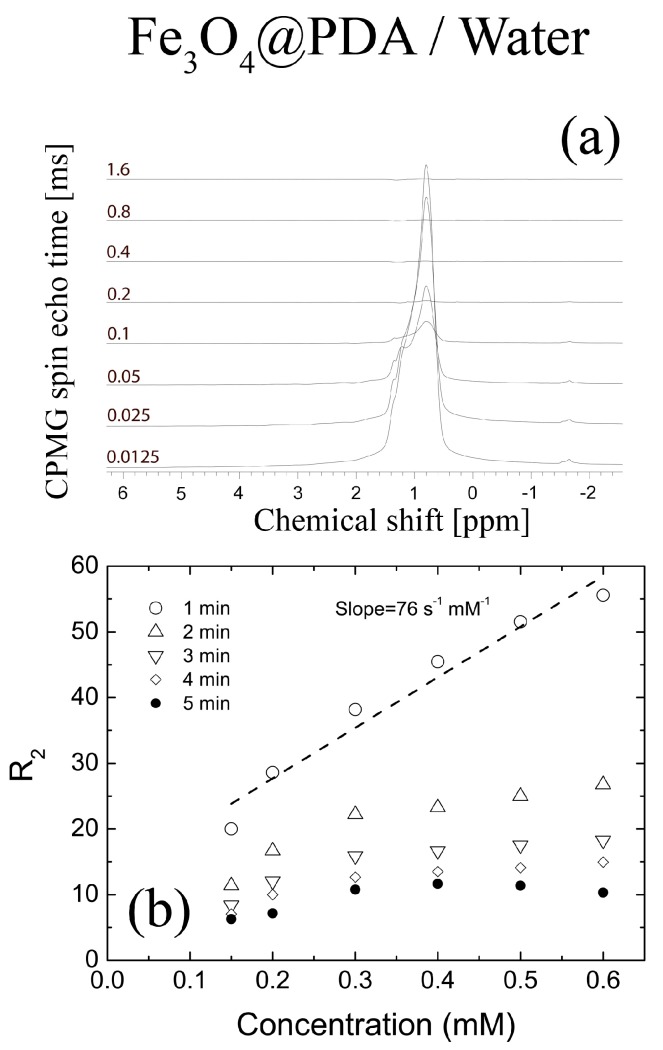
(**a**) The typical result of FT-CPMG NMR experiment for 0.15 mM Fe_3_O_4_@PDA/water sample. The relaxation time *T*_2_ was calculated using integral amplitudes of water signal for echo times, varied between 0.01 and 1.6 s with τ_cp_ = 2 ms; (**b**) The dependence of transverse relaxivities *R*_2_ on nanoparticle concentration obtained for τ_cp_ = 1 ms in bulk water solution. The fast precipitation in the strong magnetic field (high magnetic field inhomogeneity of the superconductive magnet) is a major factor affecting the correctness of the relaxation results in water suspensions. The relaxivities substantially decreased with time.

Clearly, the line widths of the water signals are highly dependent on the nanoparticle concentration. Accordingly, the relaxation rates can be estimated from line widths (FWHM) using the Bloch formula *R*_2eff_ = 1/*T*_2eff_ = (π × FWHM) [46]. The drawback of this approach is that we estimate the so-called effective relaxation rate, which includes all sorts of physical phenomena such as chemical exchange and diffusive exchange, as well as native spin-spin relaxation time [47]. The relaxation rates obtained from NMR line widths and the corresponding effective relaxation rates *R*_2eff_ from Figure 4a are summarized in Table 1.

We expect that FWHM will be dependent on the viscosity of the solvent due to diffusion exchange between the solvent environment and the vicinity of the magnetic nanoparticle. This effect can be removed from the experiment through shortening of the radio-frequency pulse (r.f. pulse) spacing time in the CPMG experiment τ_cp_ [48] A more detailed study of the transverse relaxation would require *T*_2_ dispersion experiment, where spin-echo spacing in the CPMG experiment is varied [49,50]. It should be kept in mind that the effective relaxivity *R*_2eff,_ as obtained from the NMR line widths is the limiting value of the transverse relaxation time. Accordingly, the CPMG experiment will always give lower relaxation rates due to the immanent properties of this pulse sequence, where the effects of the chemical and diffusional exchange phenomena are reversed.

Despite the analysis of the line widths, the CPMG is the most precise method of evaluation of the possible contrast properties [51]. The traditional method of performing the CPMG experiment is to obtain an envelope of spin-echo attenuation with respect to the number of 180 radiofrequency pulses. Usually, the experiment is run in a one-dimensional manner, which means that the experimental time is kept as short as possible, although at the cost of a loss of chemical shift information. Regardless, the experiment can be run in a two-dimensional manner using Fourier transform for the last spin echo for an increasing number of the r.f. pulses (iterated). The experiment lasts longer but the chemical shift information is retained. The exemplary line shapes from the Fourier Transform CPMG experiment vs the concentration of Fe_3_O_4_@PDA nanoparticles suspended in water are shown in Figure 4a. The spectra were obtained for a fixed τ_cp_ = 1 ms. The corresponding relaxation rates *R*_2_ are shown in Figure 4b. It is clear that the relaxation rates *R*_2_ are strongly dependent on the time the sample spends inside the magnet. The relaxation rates shown in Figure 4b are much lower than those shown in Table 1. First of all, the CPMG experiment can reverse the diffusion exchange process so that relaxation rates are smaller. Secondly, it is expected that *R*_2_ values will be dependent on the CPMG pulse spacing (data not shown) [50]. Thirdly, it is clear that nanoparticles precipitate in the strong magnetic field (14 T) and the experiment has to be performed very fast. Even though the data acquisition was limited down to 30 s, the obtained relaxation rates *R*_2_ were strongly time-dependent and most likely not accurate. The relaxivity for the shortest acquisition time was around 76 s^−1^·mM^−1^. In fact, this result is in agreement with the previously published report by Zheng et al., where the relaxivity value 78 s^−1^·mM^−1^ was obtained by MRI for Fe_3_O_4_@PDA dispersed in water [52]. However, in light of the performed experiments, it seems that due to strong interactions of magnetic nanostructures with strong B magnetic fields, it is nearly impossible to obtain their correct relaxation times in the water suspensions from MRI experiment alone. For instance, just to repeat our CPMG experiment, the experimental time should be kept below 1 min in total, including sample positioning and scan time.

### 3.3. Transverse Relaxation T_2_ of Water for Fe_3_O_4_@PDA and Fe_3_O_4_@PDA@SH-βCD in Agarose

**Figure 5:**
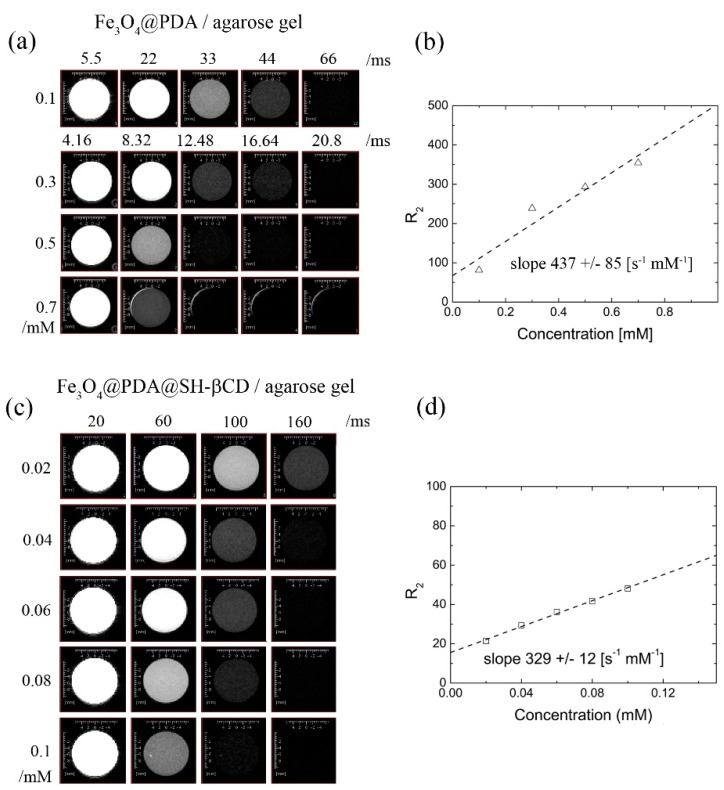
(**a**) The *T*_2_ weighted MRI images for selected spin echo times obtained for Fe_3_O_4_@PDA in 2 wt % agarose gel; (**b**) Relaxation rates *R*_2_ as well as relaxivity obtained from MRI experiment for Fe_3_O_4_@PDA in agarose gel 2 wt %; (**c**) The *T*_2_-weighted MRI images for selected spin echo times obtained for Fe_3_O_4_@PDA@SH-βCD; (**d**) The dependence of relaxation rate *R*_2_ vs. concentration for Fe_3_O_4_@PDA@SH-βCD.

In order to assess the ’live’ performance of nanoparticles, we prepared the suspensions of nanoparticles in an agarose gel (2 mg/mL). For this purpose Fe_3_O_4_@PDA and Fe_3_O_4_@PDA@SH-βCD nanoparticles were suspended in 2% agarose gel in order to avoid drift in the high magnetic field. The spin echo (MEMS) imaging results obtained for Fe_3_O_4_@PDA and Fe_3_O_4_@PDA@SH-βCD are shown in Figure 5a,c, respectively. The spin-echo-weighted MRI images were obtained for concentrations of Fe_3_O_4_@PDA varied between 0.1 and 0.7 mM. The concentrations of Fe_3_O_4_@PDA@SH-βCD were varied between 0.02 and 0.1 mM.

The calculated relaxation rates and relaxivities are shown in Figure 5b,d for Fe_3_O_4_@PDA and Fe_3_O_4_@PDA@SH-βCD, respectively. A circular Region of Interest (ROI) was selected for each sample (10 mm plastic vials). The intensities were exported and *T*_2_ relaxation times were calculated and shown in Figure 5b–c. We did not subtract the agarose relaxation rate from our data since it does not affect the slope. This can be done by using the following equation: *R*_total_ = *R*_particles_ + *R*_agarose_. The relaxivity of the Fe_3_O_4_@PDA suspended in agarose matrix is much higher than one obtained for a suspension of nanoparticles in water (437 vs. 76 s^−1^·mM^−1^). The reason for this is that the gel prevents the nanoparticles from diffusing and precipitating due to the strong magnetic field. At the same time the water diffusion is inhibited due to the porous structure of a hydrogel, therefore, a restricted diffusion should be observed [53]. Furthermore, this value is higher than previously reported [52]. All in all, we believe that the relaxivity results obtained in agarose gel phantoms are free from the precipitation effects, and therefore it is reasonable to expect higher relaxivity values. The relaxivity obtained for Fe_3_O_4_@PDA nanoparticles was around 437 mM^−1^·s^−1^. The addition of the SH-βCD layer decreased the relaxivity down to 329 mM^−1^·s^−1^. Comparatively, the relaxivity obtained for a high-performance *T*_2_ contrast agent, such as octapod iron oxide nanoparticles, is higher, reaching relaxivity at values as high as 680 mM^−1^·s^−1^ [54]. 

In turn, previously reported relaxivities of magnetic clusters covered with PDA are characterized by lower relaxivities as high as 230.5 and 78.1 mM^−1^·s^−1^, respectively [52,55]. It is most likely that the results reported by other authors were obtained in water suspensions and might be prone to precipitation in the high magnetic field. Before the NMR sample tube is settled in the probe, it passes the strong magnetic field gradient of the superconductive magnet. During that phase, the precipitation process is most likely initiated and the smaller values of relaxivities are underestimated. All in all, the superparamagnetic nanoparticles usually affect the transverse relaxation rate *R*_2_ = 1/*T*_2_, rather than *R*_1_. Accordingly, the current contribution was focused on the potential ability of Fe_3_O_4_@PDA and Fe_3_O_4_@PDA@SH-βCD nanoparticles to shorten the transverse relaxation *T*_2_.

### 3.4. NIR Laser Irradiation

**Figure 6:**
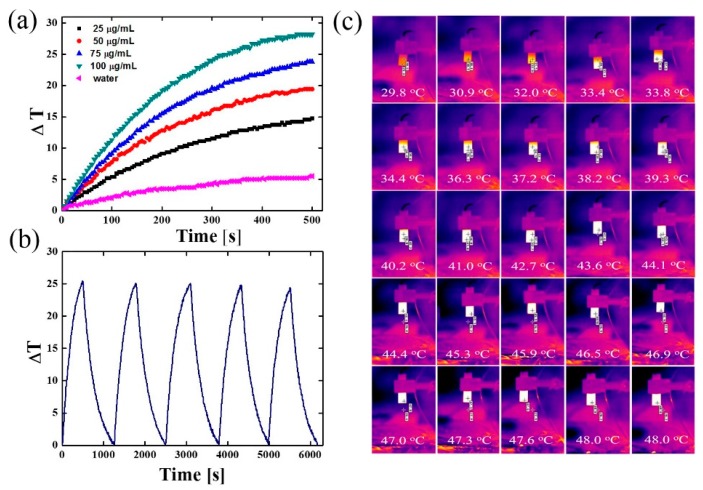
(**a**) Temperature change versus time, recorded for nanomaterial B at various concentrations; (**b**) Photostability of nanomaterial B at concentration 100 µg/mL under beam NIR of 2 W/cm^2^; (**c**) Infrared thermal images of Fe_3_O_4_@PDA nanoparticles upon irradiation with NIR laser (808 nm, 2.0 W/cm^2^). Time interval 20 s.

Polydopamine is known to possess strong absorption in the NIR (near-infrared) region, and because of this it has been used recently as a photothermal agent in cancer therapy. Thus, we investigated the absorption of NIR light by nanomaterial B to see whether they could be considered as efficient photothermal agents. In the performed experiments, nanoparticles B were irradiated with a laser beam of 808 nm at power 2 W/cm^2^ for 500 s at increasing concentrations, which caused a large temperature change in the medium (see Figure 6a–c). 

As expected, the temperature change increased, while the concentration of nanoparticles in sample B also increased. Nevertheless, the obtained nanomaterial exhibited high photothermal properties even at as low concentrations of nanomaterial as 25 µg/mL, since the medium temperature was increased almost about 15 °C under NIR irradiation conditions. Further increases in concentration to 50 and 75 µg/mL resulted in raising the temperature up to 20 and 23 °C, respectively. At a concentration of 100 µg, the medium temperature was elevated almost 30 °C. This phenomenon took place because, together with an increased mass of nanomaterials, we increased the content of PDA, which is most responsible for photothermal properties. (See Supplementary Materials Figures S7 and S8). It is important to stress that irradiation of pure water, with the same laser power, did not significantly influence water temperature. The temperature change versus time is presented in Figure 6a. Furthermore, the photostability test of material B showed that they could be used in at least 5 on/off cycles of laser irradiation, which proved their high photothermal stability (Figure 6b). 

### 3.5. Drug Loading and Release

An important issue in drug delivery using nanomaterials is to know the level of drug loading on a nanocarrier and its release profile. Thus, we used doxorubicin as a model chemotherapeutic drug, to determine the loading capacity of the material B. The loading tests were carried out at room temperature for 24 h in PBS buffer at pH 7.4 in order to prevent DOXO degradation and assure the optimal conditions for its encapsulation. Afterwards, the amount of loaded drug was evaluated by UV-Vis spectroscopy using the standard calibration curve method. The determined loading was ~900 µg/mg which corresponded to an encapsulation efficiency of 90%. As a result, we obtained Fe_3_O_4_@PDA@SH-βCD@DOXO with a higher loading of doxorubicin than recently reported [30].

In the next step, the drug release profile was determined by the incubation of Fe_3_O_4_@PDA@SH-βCD@DOXO with citric buffer at pH 4.5 and 5.5, respectively. The cumulative release of doxorubicin within 10 h is around 9% and 11% for pH 5.5 and 4.5, respectively (see Supplementary Materials Figure S9). The maximum doxorubicin release was achieved in both cases after almost 50 h, and was 9% and 10% for pH 5.5 and 4.5, respectively. This release profile indicates that our nanomaterials can release the drug in a slow and sustained way, which is a desirable feature in potential cancer treatments. Since cancer cells are known to have lower pH because of high glycolytic activity, we assume that DOXO can be unloaded inside cancer cells without causing severe side effects during its delivery by nanocarriers to the tumoral cells since only 2% of the drug was released in PBS buffer at pH 7.4 after 48 h. This shows that the drug was not extensively released in the physiological pH, but was released in a pH-dependent manner in an acidic environment. In addition to all these, the nanomaterial showed improved chemical stability in acidic conditions in comparison to recently reported PDA-coated magnetic nanoparticles since we did not observe any signs of carrier degradation [30].

### 3.6. Activity of Nanomaterials in Anticancer Therapy

**Figure 7:**
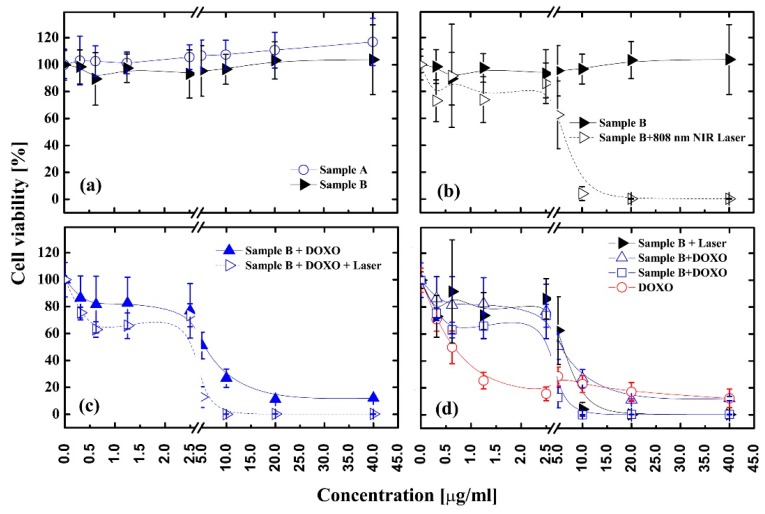
Cell viabilities of HepG2 cells after being incubated with different nanomaterials at various concentrations. (**a**) Sample A—Fe_3_O_4_@PDA and Sample B—Fe_3_O_4_@PDA@SH-βCD; (**b**) Sample B—with and without laser irradiation; (**c**)Sample B—loaded with DOXO with and without laser irradiation; (**d**) Replotted data from (**b**,**c**) plus free DOXO as the reference.

**Figure 8:**
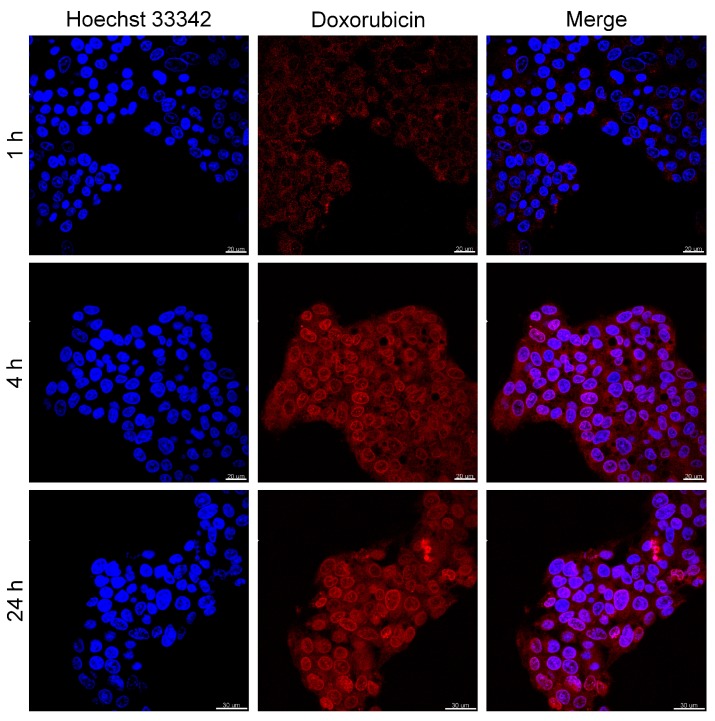
Confocal microscopy images of HepG2 cells incubated with Fe_3_O_4_@PDA@SH-βCD@DOXO at different time points. (**1st row**) blue-nuclei stained with Hoechst 33342; (**2nd row**) Red-DOXO fluorescence; (**3rd row**) represents column one and two combined.

The cytotoxicity and activity analysis in the anticancer therapy of liver cancer cells of the obtained nanomaterials were performed using WST assay. The results are shown in Figure 7. In this test, nanoparticles A and B in concentrations up to 40 µg/mL were cultivated with HepG2 for 24 h in standard conditions at 37 °C. The obtained results showed that neither nanomaterials caused any significant toxicity on the cancer cells in the applied concentration range, since almost no changes in the cells’ viability were observed. Since our particles showed strong photothermal properties (Figure 6a,b), we decided to investigate the efficacy of PTT on HepG2 cells using Fe_3_O_4_@PDA@SH-βCD). The cell viability results after irradiation of sample Fe_3_O_4_@PDA@SH-βCD with NIR light are shown in Figure 7b. It is worth highlighting that the cancer cells were a priori incubated with Fe_3_O_4_@PDA@SH-βCD for 4 h and then cells were irradiated with a laser beam of 2 W/cm^2^ for 5 min. As can be seen in Figure 7b, PTT was not efficient at low nanomaterial concentrations below 2.5 µg/mL, and the cells’ viability was above 80%. Apparently, a much stronger PTT effect was observed for concentrations higher than 5 µg/mL up to 40 µg/mL. At those concentrations, the cells’ viability decreased from 60% to almost 0%, demonstrating that higher concentrations were necessary to generate the hyperthermic effect. However, it should be pointed out that bulk doxorubicin at a concentration up to 5 µg/mL was more efficient in killing cancer cells than the assays performed with PTT on Fe_3_O_4_@PDA@SH-βCD, since the cells’ viability decreased down to 15%. We also investigated the effect of irradiation of cancer cells with laser beam. We observed that cells directly irradiated with NIR beam suffer from increased cytotoxicity compared to those outside the beam as proven by LIVE/DEAD assay (see Supplementary Materials Figure S10). Furthermore, we cultivated HepG2 cells with Fe_3_O_4_@PDA@SH-βCD loaded with DOXO to investigate its chemotherapeutic behavior and to investigate speed of internalization of nanomaterial (see Figure 7c and Figure 8). At low nanomaterials concentration, the efficacy of drug delivery was not as robust, because the cells’ viability did not decrease as significantly as expected. Also, a concentration range up to 5 µg/mL of Fe_3_O_4_@PDA@SH-βCD@DOXO was not so active as bulk DOXO.

The simultaneous application of CT-PTT at a concentration of 5 µg/mL exhibited a better therapeutic outcome in comparison to bulk doxorubicin (see Figure 7d). Furthermore, at 5 µg/mL concentration, the combined CT-PTT caused a difference of 39% in cells’ viability in comparison to PTT treatment alone. Although, when the concentration of nanomaterial B was increased above 5 µg/mL, the differences between combined chemo- and photothermal therapy and sample B bearing DOXO are smaller but still significant. It is 25%, 11%, and 12% at 10, 20, and 40 µg/mL, respectively. The higher performance of both sample B and sample B + DOXO above 5 µg/mL can be explained by the fact that our materials were nanosized objects, therefore their uptake was higher than free DOXO. This drug is hydrophobic and therefore its combination with nanoparticles enhanced the solubility and more efficient delivery to cancer cells. 

## 4. Conclusions

We have applied the Fe_3_O_4_@PDA@SH-βCD nanoparticles loaded with doxorubicin in combined chemo- and photothermal therapy. Polydopamine-coated magnetic nanoparticles modified with 6-thio-β-cyclodextrin were prepared and investigated by means of TEM, SQUID, FT-IR, XPS, and MRI. The performed analyses proved the modification of nanoparticles with cyclodextrins via the covalent C–S bond. Synthesized nanoparticles A and B were found to be nontoxic and can be characterized by strong photothermal properties. Additionally, the prepared systems are characterized by a high drug-loading capacity (doxorubicin) and showed enhanced performance in the combined chemo- and photothermal therapy of liver cancer cells at low nanoparticle concentrations. In addition, both Fe_3_O_4_@PDA and Fe_3_O_4_@PDA@SH-βCD nanoparticles turned out to be robust *T*_2_ contrast agents for MRI. It is worth highlighting that the performed experiments showed that immobilization of nanoparticles in agarose gel significantly influences their contrast properties in comparison to their water dispersion, in which they there are not stable when a magnetic field is applied. Therefore, our protocol assures new insight in measurements of MRI contrast properties of PDA-coated nanostructures. Overall, this multimodal nanoplatform has promising potential as an effective therapeutic agent for multitasking cancer treatment as proven during in vitro studies. Furthermore, our results are of great importance in the field of synthesis and application of multifunctional magnetic nanoparticles based on cyclodextrins and polydopamine, shedding new light on their application in advanced anticancer therapy. 

## Figures and Tables

**Table 1:** The dependence of the ^1^H nuclear magnetic resonance line widths vs of nanomaterial B and effective relaxation rates *R*_2eff_ as obtained from the line widths.

Concentration [mM]	FWHM [Hz]	*R*_2eff_ [s^−1^]
0.15	240	754
0.20	252	792
0.30	353	1108
0.40	357	1121
0.50	370	1162
0.60	380	1193

## Supplementary Materials

The following are available online at http://www.mdpi.com/2079-4991/8/3/170/s1.
