# Supplementary material for: Cyclodextrin-Based Magnetic Nanoparticles for Cancer Therapy
Source: Nanomaterials (Basel). 2018 Mar 16;8(3):170. doi: 10.3390/nano8030170 (PMC5869661; doi:10.3390/nano8030170)
Supplement: Supplementary file 1 [file nanomaterials-08-00170-s001.pdf]

*Article*

# Cyclodextrin-Based Magnetic Nanoparticles for Cancer Therapy

**Radosław Mrówczyński [1,*], Artur Jędrzak [1,2], Kosma Szutkowski [1,*], Bartosz F. Grześkowiak [1], Emerson Coy [1], Roksana Markiewicz [1], Teofil Jesionowski [2] and Stefan Jurga [1]**

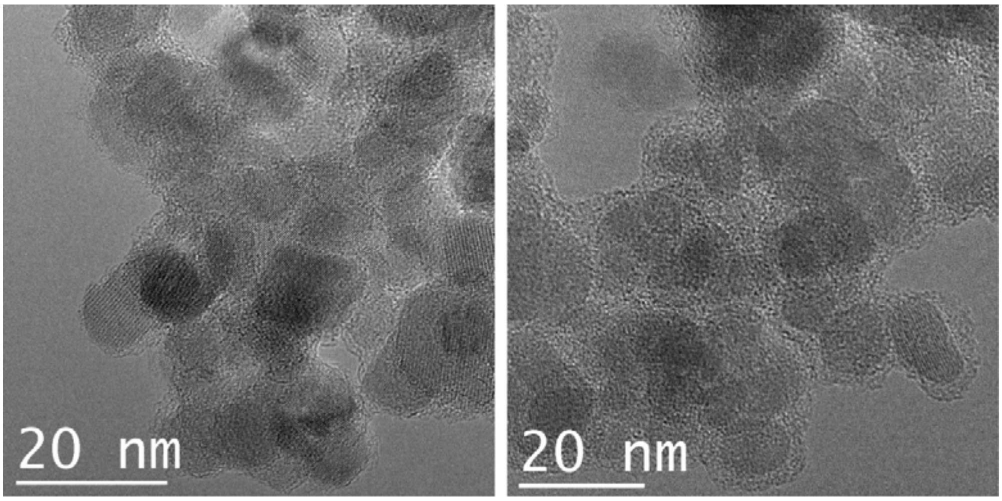


**Figure S1**. HR-TEM pictures of nanomaterial B with visible crystalline lattice.

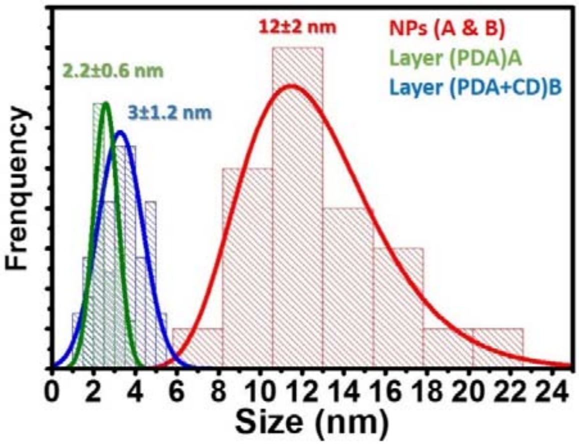


**Figure S2**. PDA layer thickness distribution in sample A (Green color) and B (Blue color) and size distribution of magnetic core in both samples (Red color). Individual particles were measured using DigitalMicrograph software from HR-TEM images. A collection of 200 individual measurements was collected and presented in histogram form.

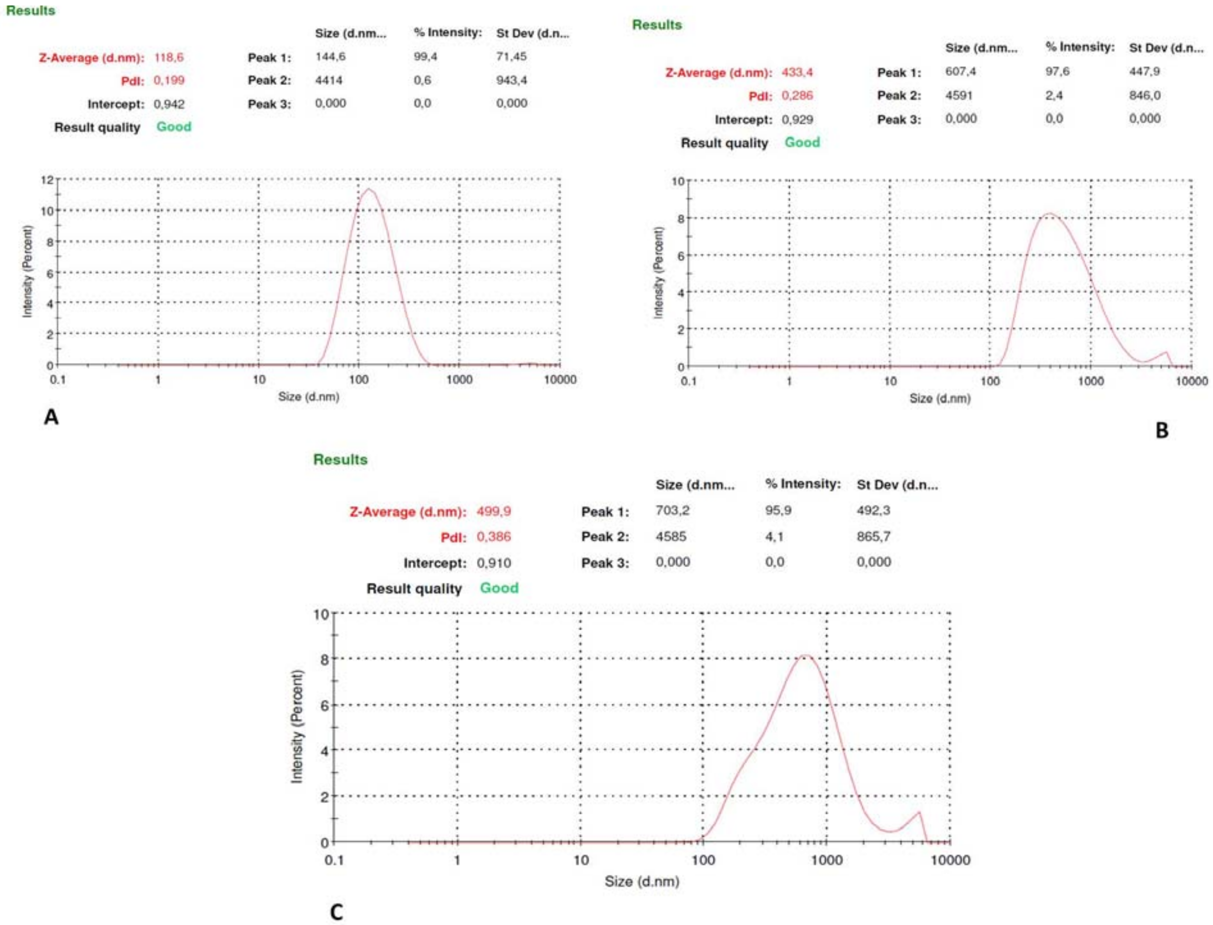


**Figure S3.** Hydrodynamic diameter recorder for bare magnetite nanoparticles (**A**); $Fe_3O_4$@PDA (**B**) and $Fe_3O_4$@PDA@SH-βCD (**C**).

**Table S1.** Changes of hydrodynamic diameter in time recorder for $Fe_3O_4$@PDA@SH-βCD

| Day | Hydrodynamic diameter (nm) |
|---|---|
| 0 | 500 |
| 3 | 683 |
| 6 | 684 |
| 7 | 684 |

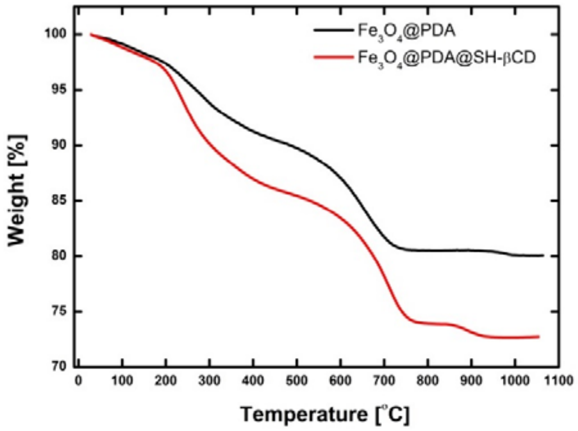


**Figure S4.** TGA curves for $Fe_3O_4$@PDA and $Fe_3O_4$@PDA@SH-βCD.

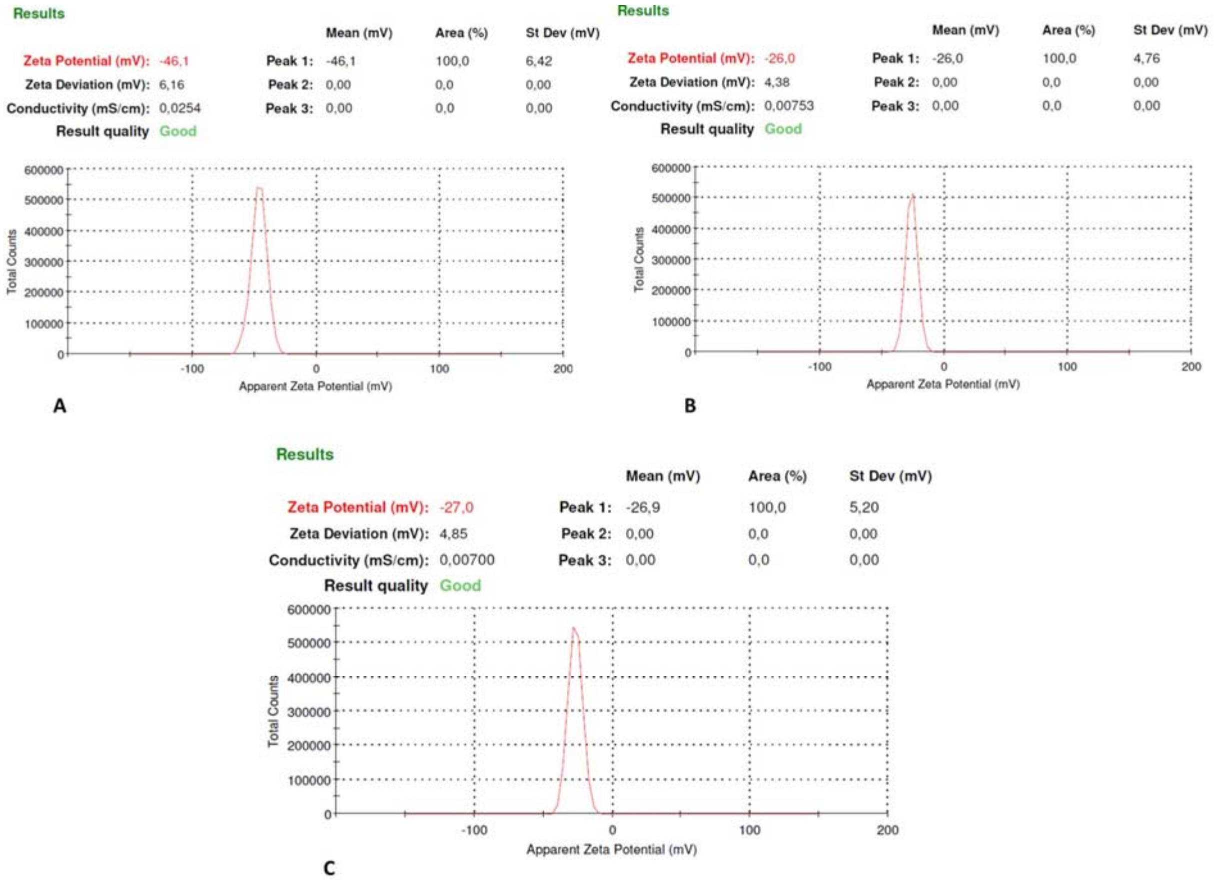


**Figure S5.** Zeta potential values recorder for bare magnetite nanoparticles (**A**); $Fe_3O_4$@PDA (**B**) and $Fe_3O_4$@PDA@SH-βCD (**C**).

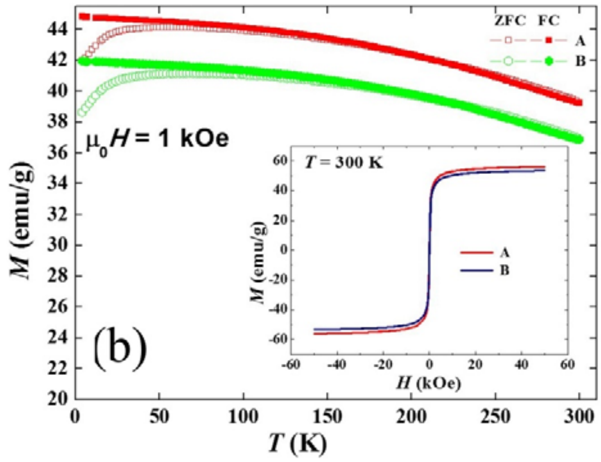


**Figure S6.** Magnetic properties of sample A and B.

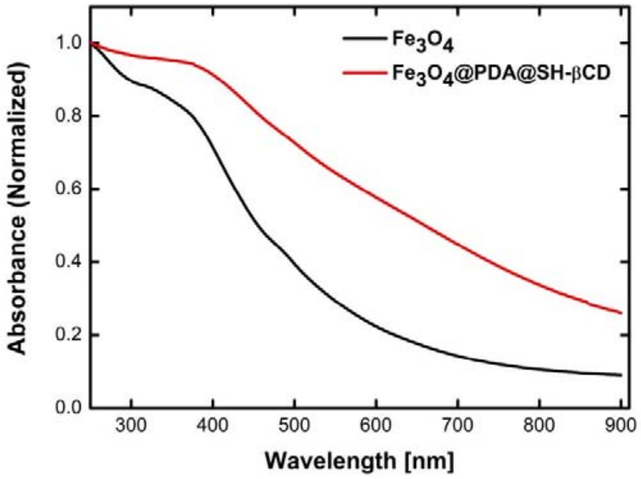


**Figure S7.** Absorption spectra of magnetic nanoparticles and nanomaterial B in range from 250 to 900 nm.

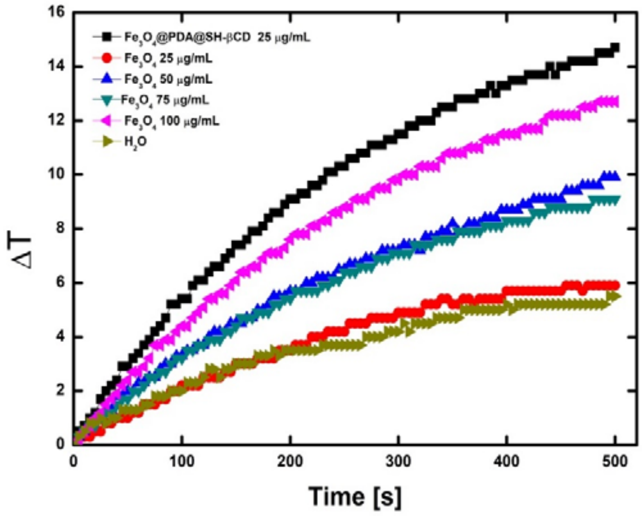


**Figure S8.** Temperature changes vs. time caused by bare magnetite at different concertation and nanomaterial B at concertation of 25 μg/mL.

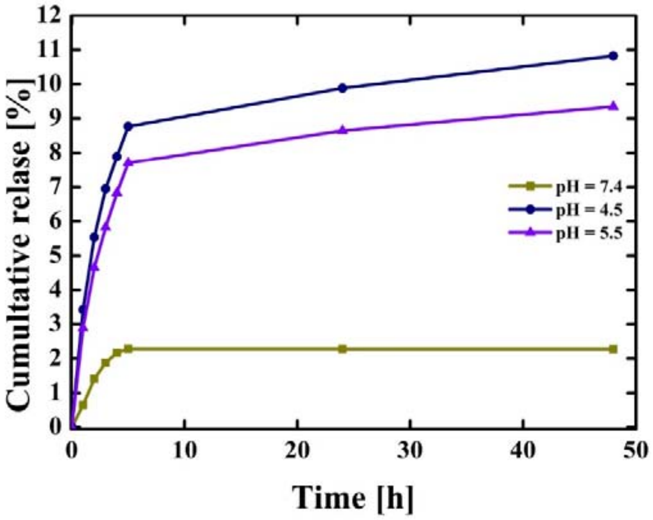


**Figure S9.** DOX release profile under various pH from sample B + DOX.

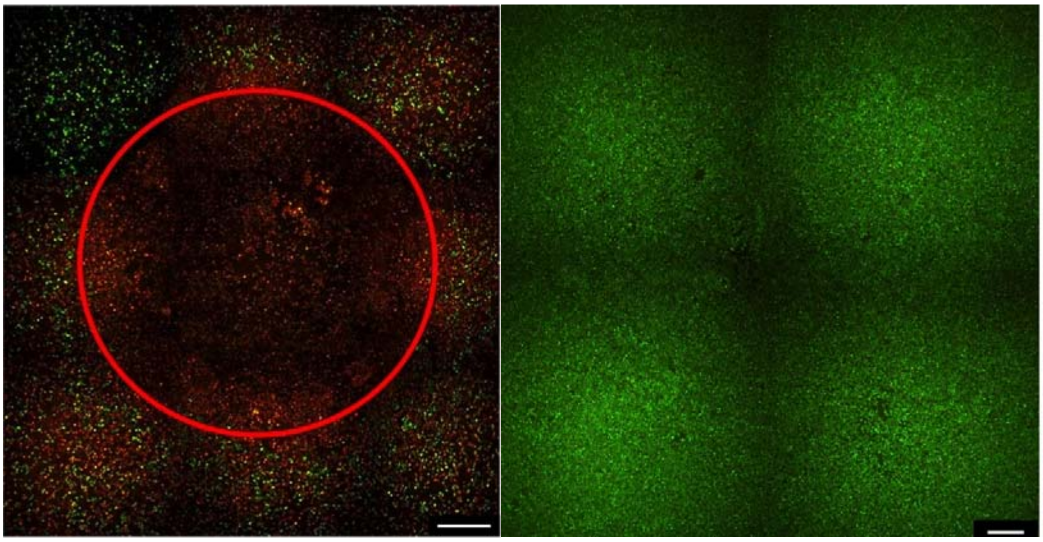

(a) (b)

**Figure S10.** Figure (**a**) The results of LIVE/DEAD after irradiation of HepG2 cell priori incubated with nanomaterials B at concertation 40 μg/mL. Red-death cell, Green-live cells; Figure (**b**) HepG2 cells irradiated with laser beam without nanomaterial B. Green-live cells. Scale bar 500 μm.
